# Supplementary material for: Lichen Biodiversity and Near-Infrared Metabolomic Fingerprint as Diagnostic and Prognostic Complementary Tools for Biomonitoring: A Case Study in the Eastern Iberian Peninsula
Source: J Fungi (Basel). 2023 Oct 31;9(11):1064. doi: 10.3390/jof9111064 (PMC10672448; doi:10.3390/jof9111064)

Healthy ID 1

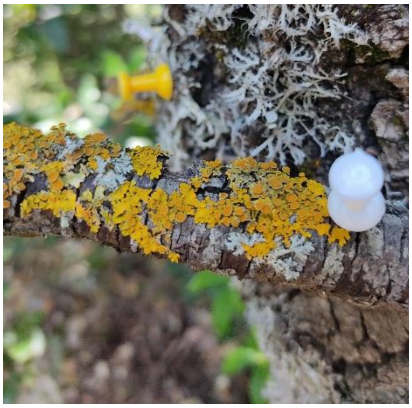

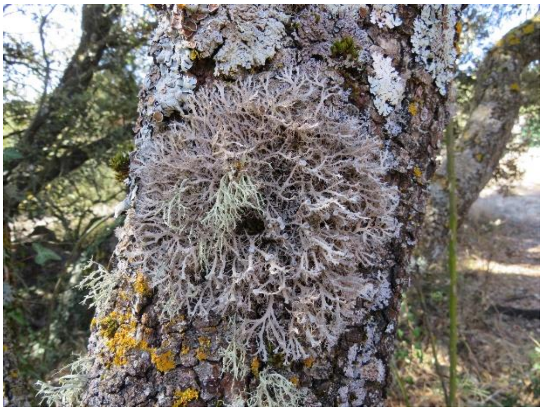

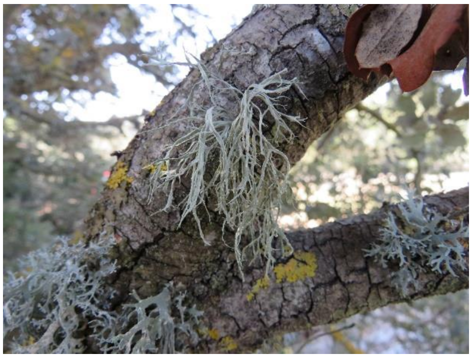

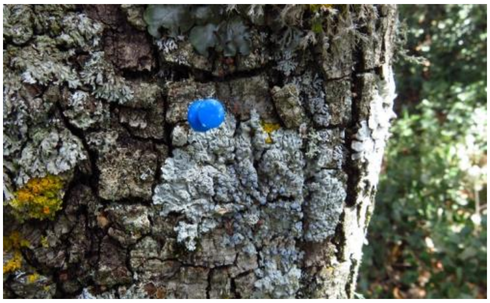

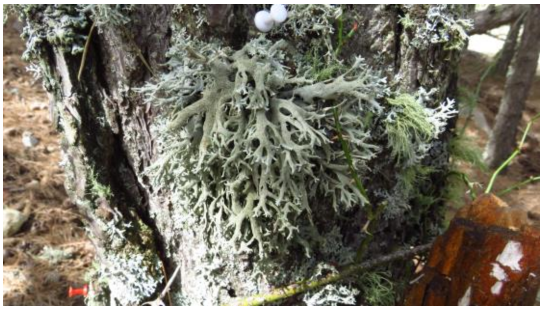

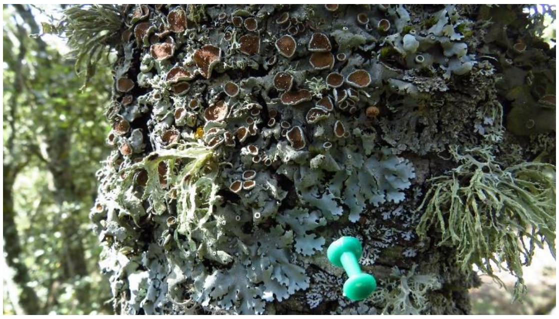

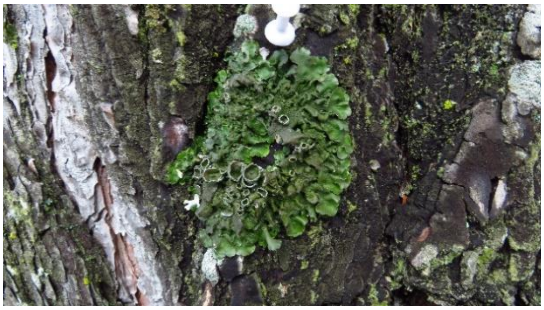

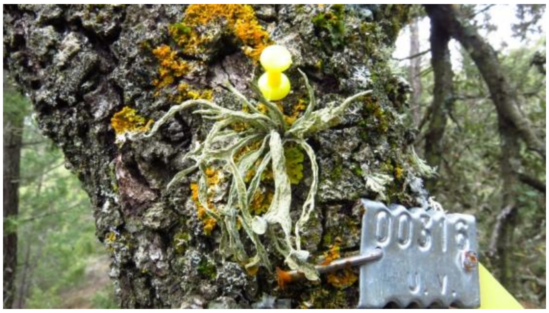

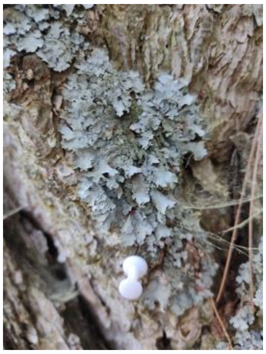

Appearance of new lobules ID4

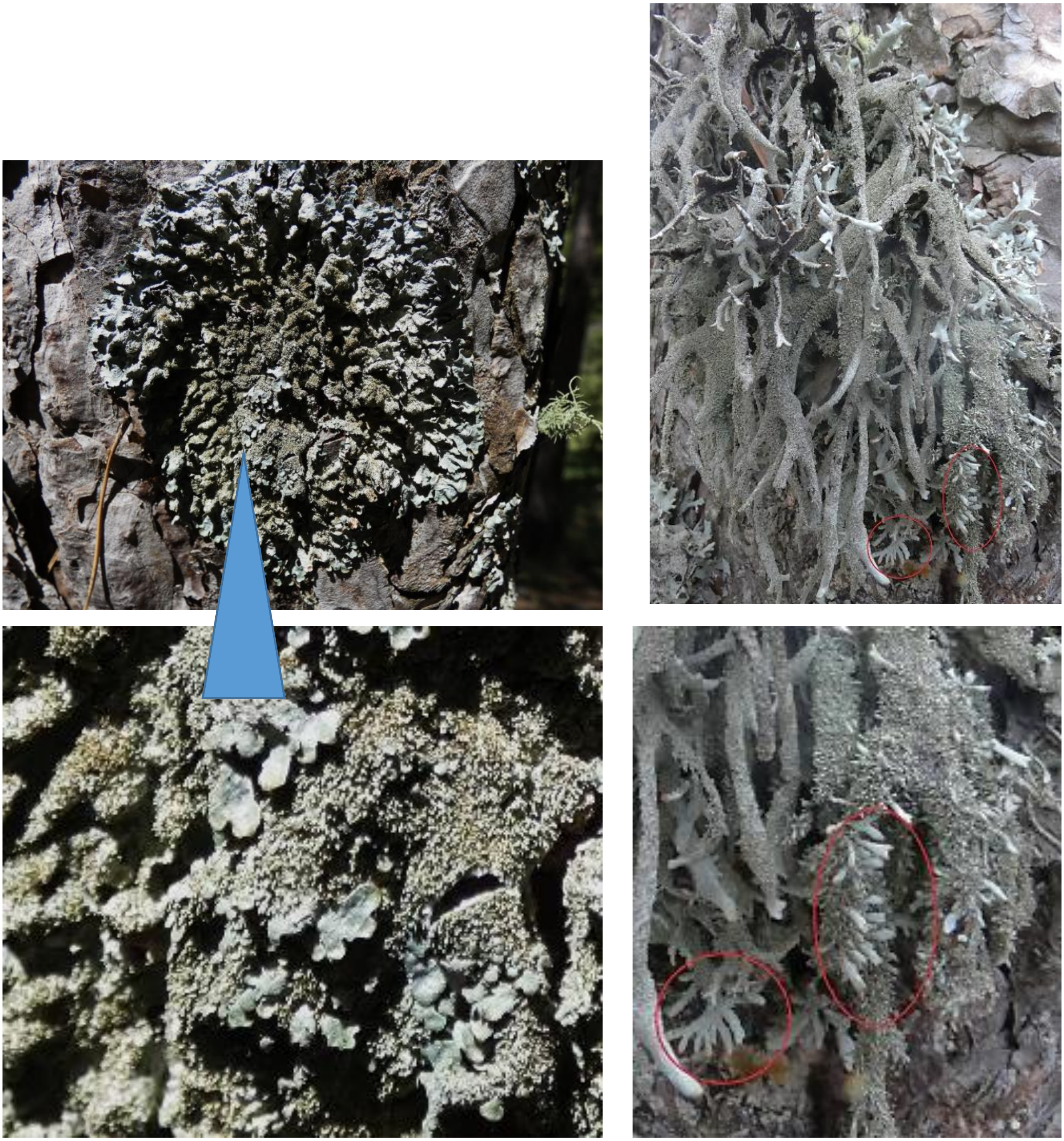

Absence of thallus

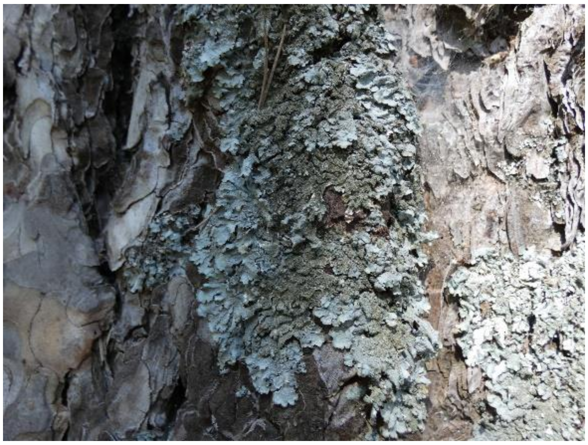

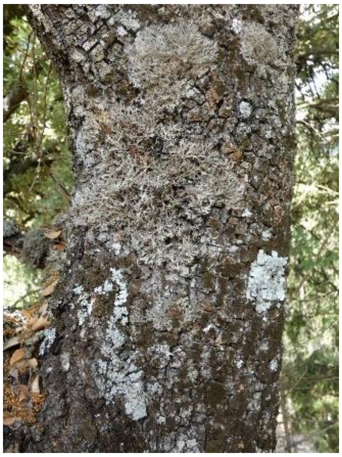

0-20% ID2

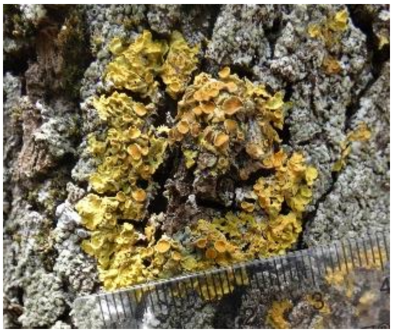

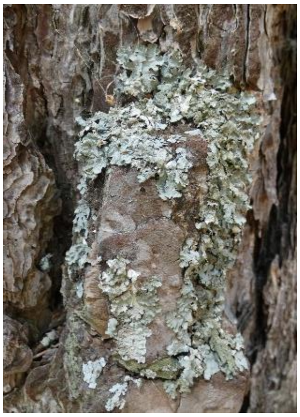

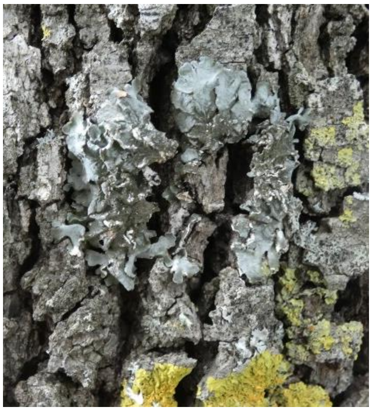

20-50% ID3

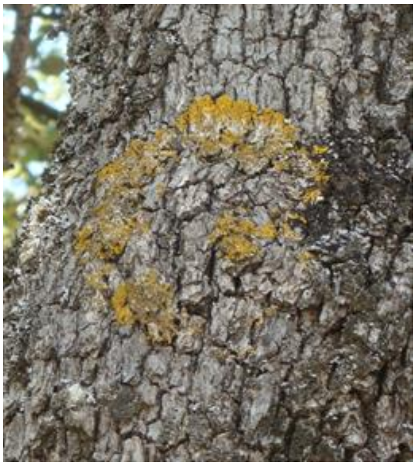

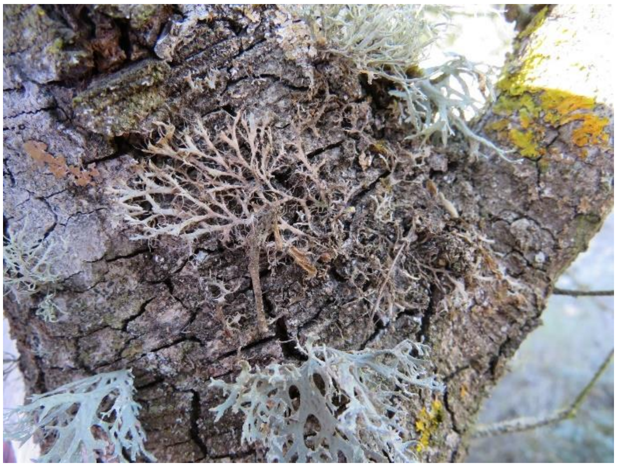

50-75% ID4

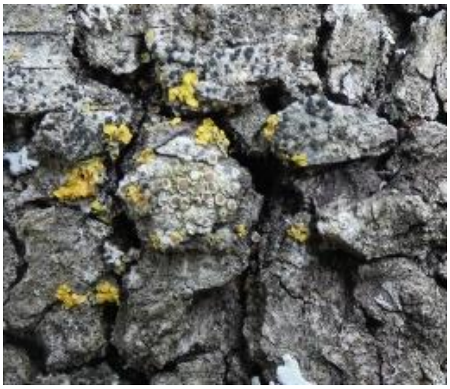

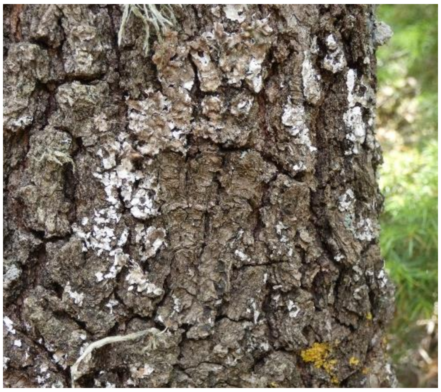

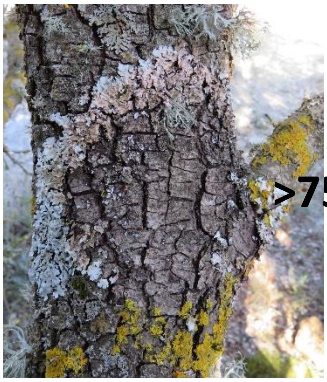

>75% ID5

Lack of cortex/necrosis

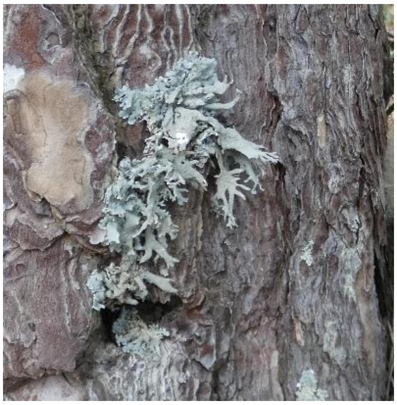

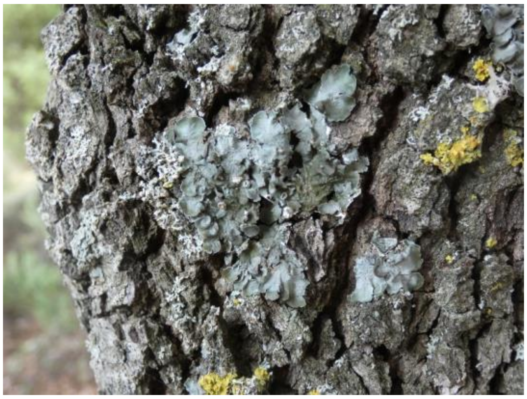

0-10% ID2

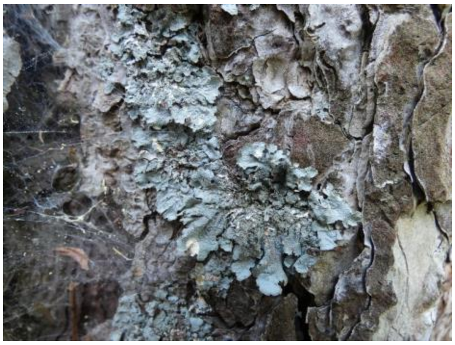

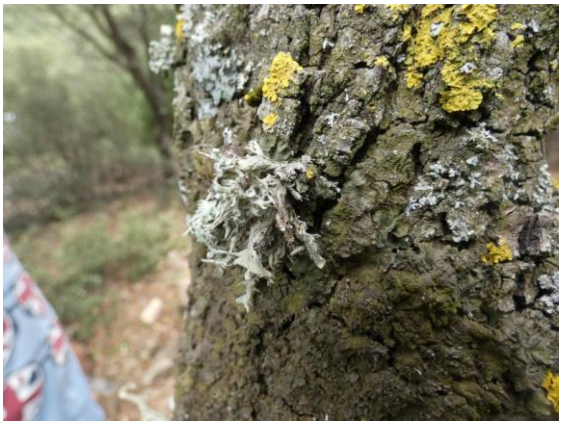

10-50% ID3

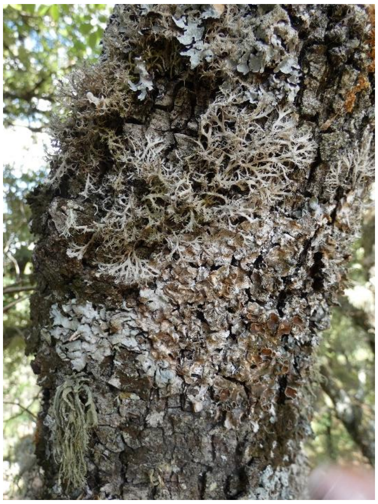

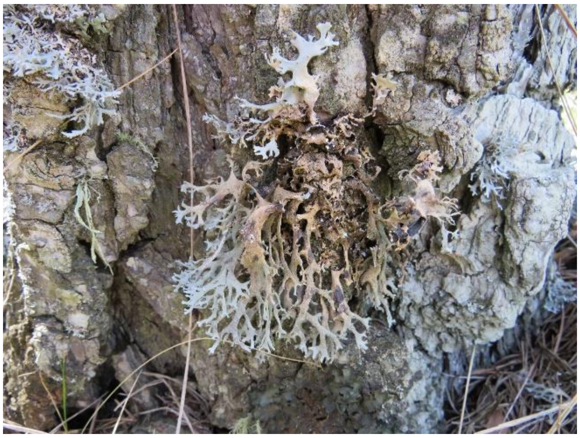

50-75% ID4

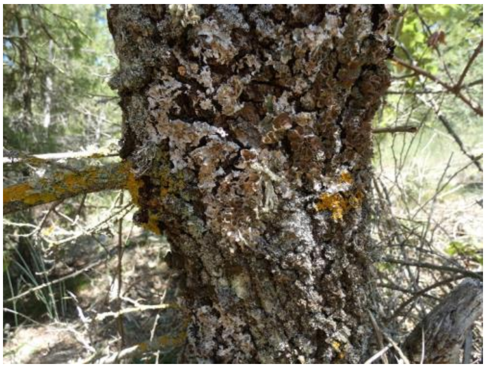

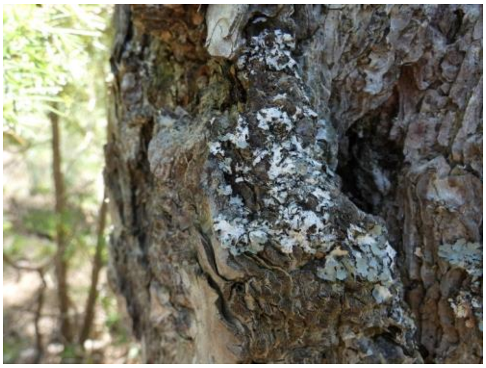

>75% ID5

Stains

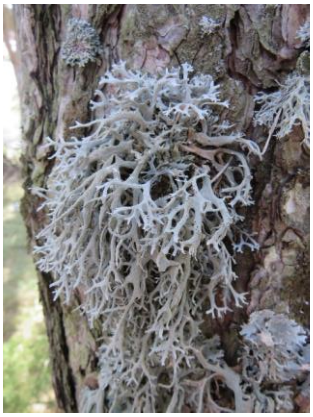

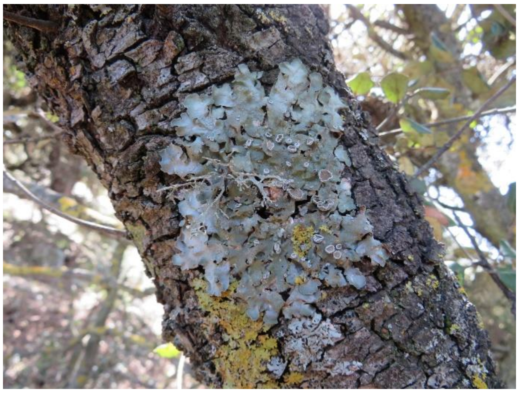

0-20% ID2

20-50% ID3

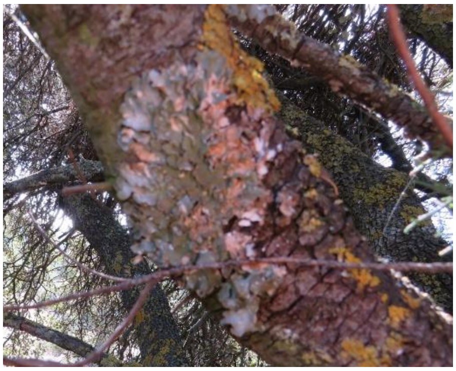

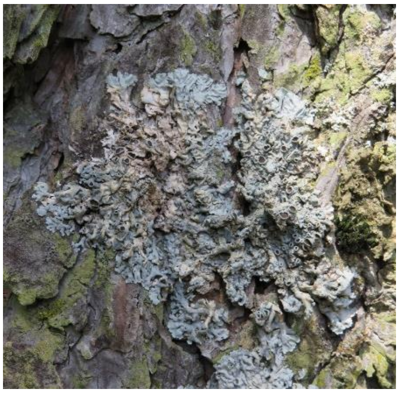

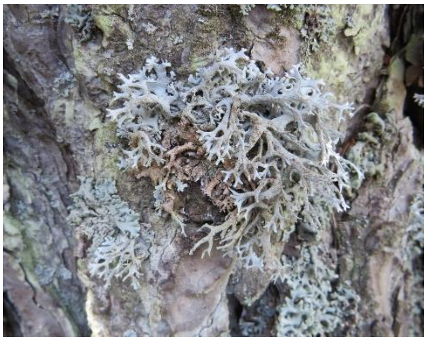

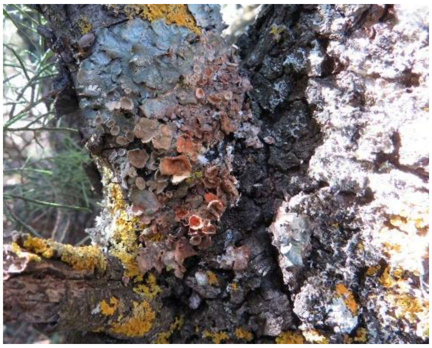

50-100% ID4

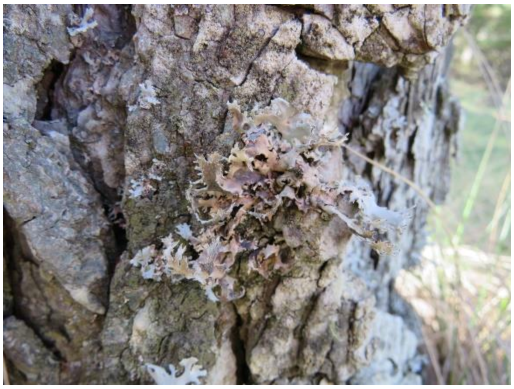

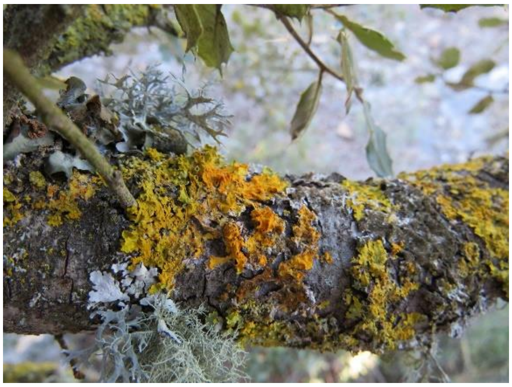

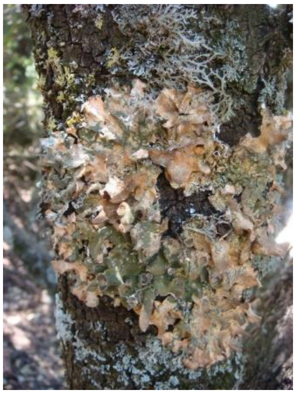

Parasites ID2/ID3

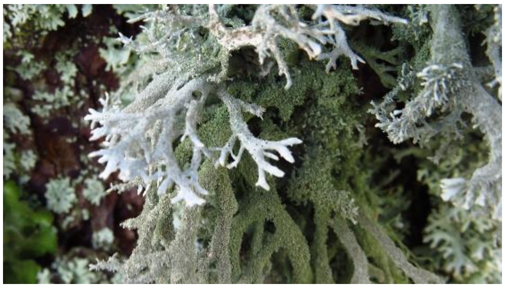

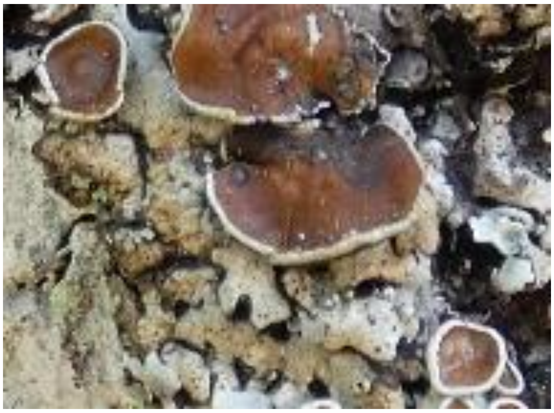

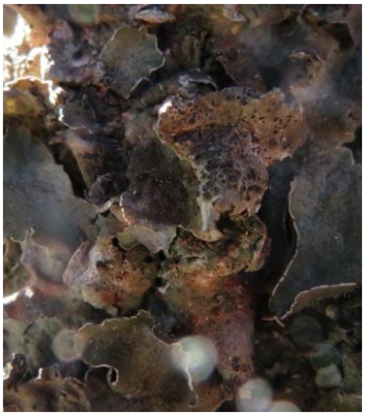

Morphological alterations ID2

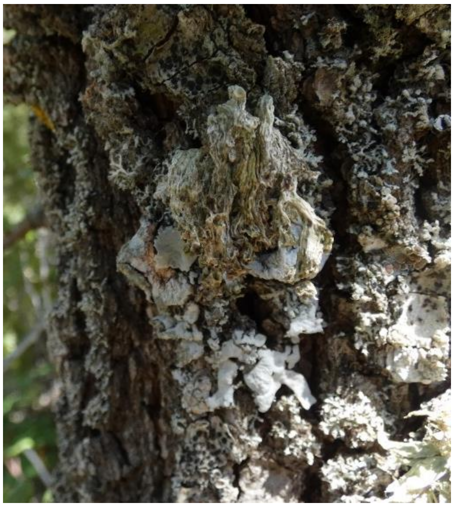

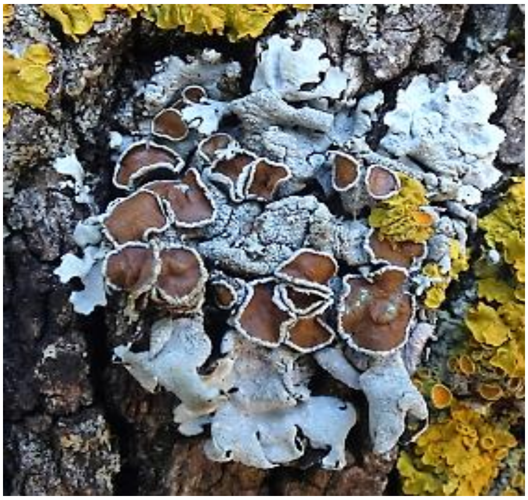

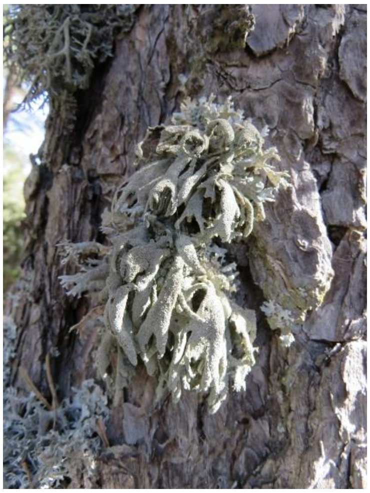

Thallus deformation

Twisting of lobules

Dark central stain ID3

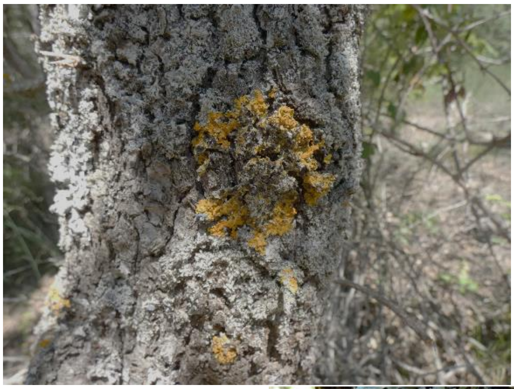

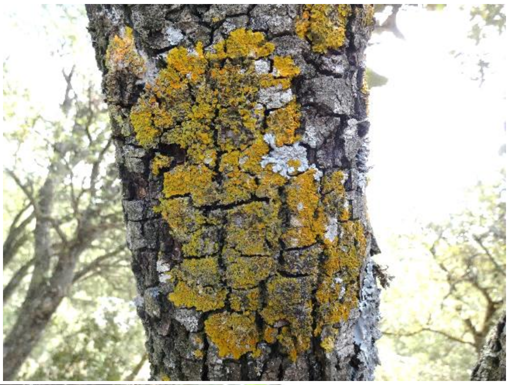

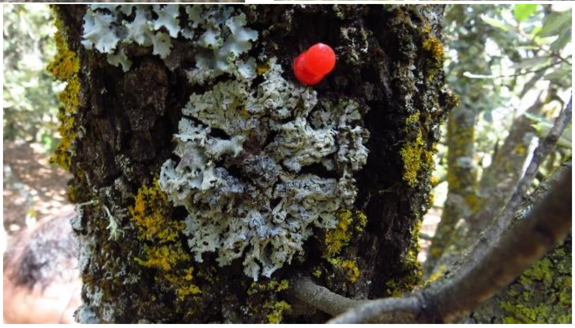

Excessive reproductive structures ID2

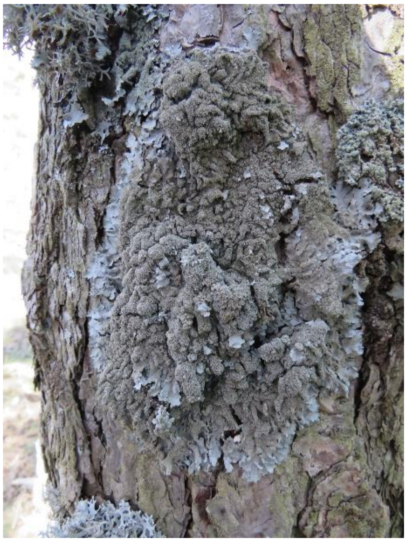

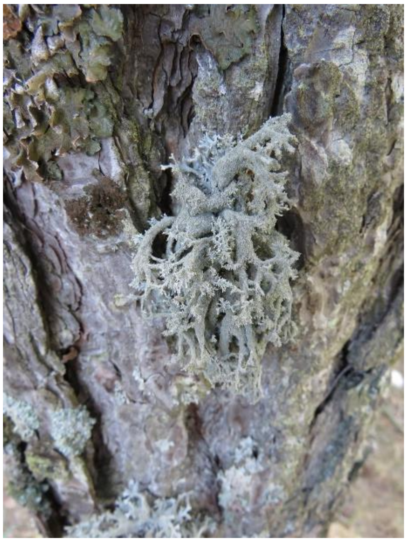

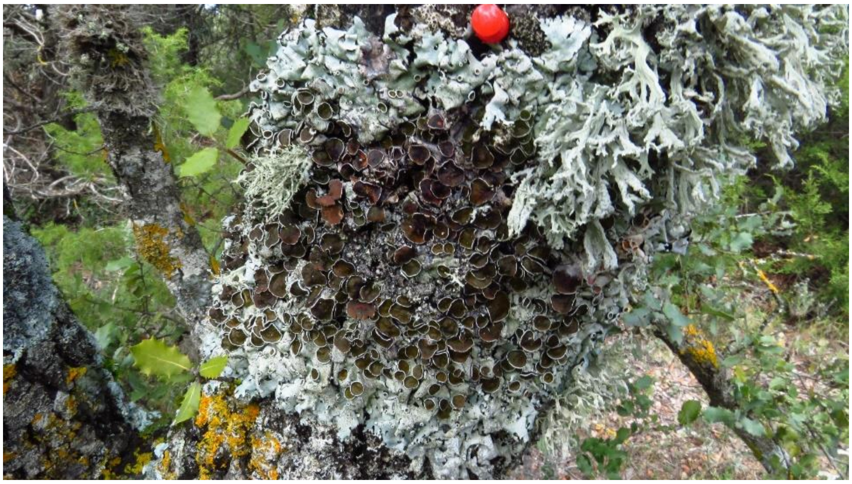

Dead thallus ID5

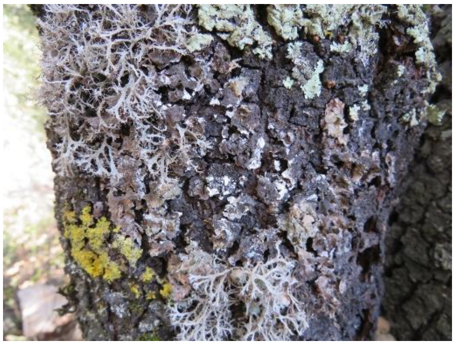

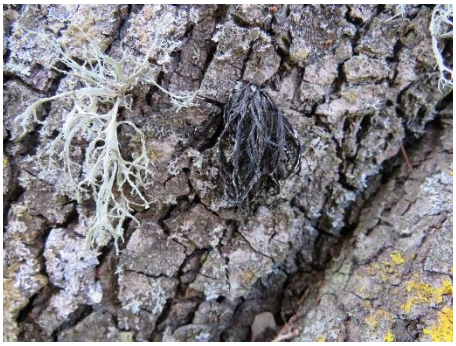

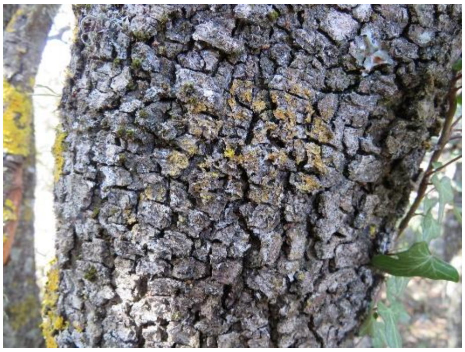

Supplement: Supplementary file 1 [file jof-09-01064-s001.zip › Figure S1.pdf]
