# Supplementary material for: Lichen Biodiversity and Near-Infrared Metabolomic Fingerprint as Diagnostic and Prognostic Complementary Tools for Biomonitoring: A Case Study in the Eastern Iberian Peninsula
Source: J Fungi (Basel). 2023 Oct 31;9(11):1064. doi: 10.3390/jof9111064 (PMC10672448; doi:10.3390/jof9111064)
Supplement: Supplementary file 1 [file jof-09-01064-s001.zip › supplementary.pdf]

Supplementary material

Figure S1. Grid used in 1997 and 2022 campaigns to measure the Index of Atmospheric Purity.

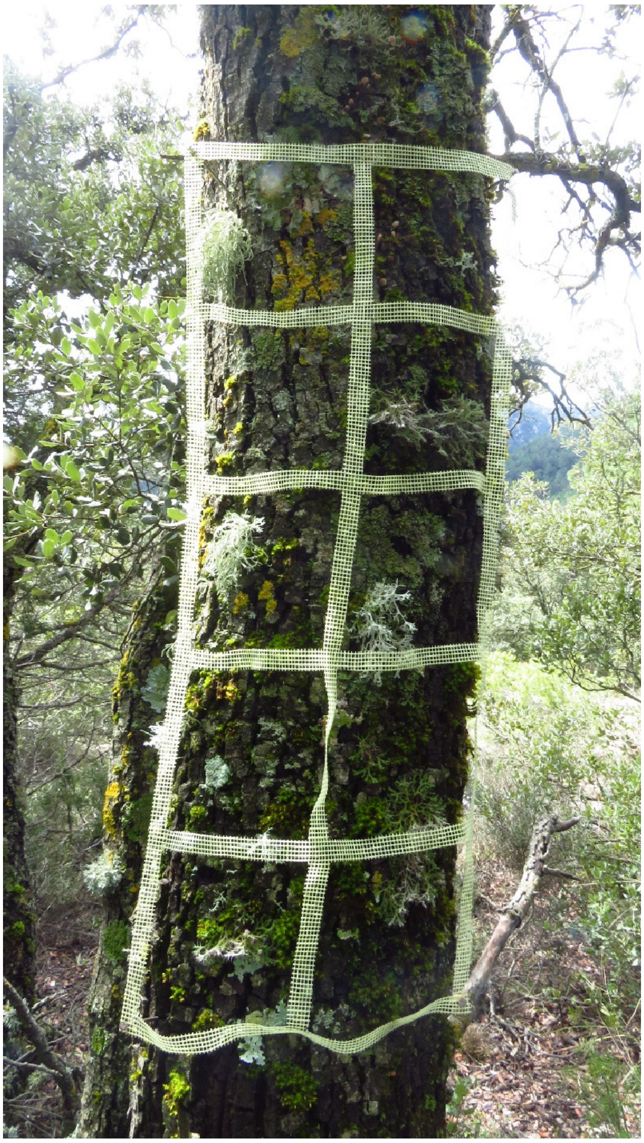

Figure S2. Visual example found in the field of different ranges of Damage Index for selected macrolichen species

ZIP file S1: Original data and scripts for the analysis of IIndex of Atmospheric Purity and Damage Index.

Table S3: Template for recording damage index used in the field

DI	visual damage
1	Healthy
2	Stains 0-20%
2	Excessive reproductive structures
2	Parasites 0-50%
2	Absence of thallus 0-20%
2	Morphological alterations:twisting of lobules, bulging of the thallus
2	Lack of cortex/necrosis 0-10%
3	Stains 20-50%
3	Parasites 50-100%
3	Absence of thallus 20-50%
3	Dark central stain
3	Lack of cortex/necrosis 10-50%
4	Stains 50-100%
4	Absence of thallus 50-75%
4	Lack of cortex/necrosis 50-75%
4	Appearance of new lobules
4	Fragile thallus
5	Absence of thallus >75%
5	Lack of cortex >75%
5	Dead thallus

Table S4: Mean Index of Atmospheric Purity (IAP) values for each locality and year. Also Student's T test between years and normality of residuals Shapiro test.

**Locality**	**IAP.1997**	**IAP.2022**	**t.test.p**	**Shapiro.p**
Bojar	35.33	18.5	0.039	0.39
Cinctorres	62.00	20.4	5.7e-08	0.29
Collado Gavilan	38.10	25.8	0.0091	0.47
Corachar	3.90	7.7	0.0051	0.44
Toro-*Pinus*	41.20	17.5	1e-06	0.028
Toro-*Quercus*	73.80	29.7	0.00086	0.41
Villarroya Pinares	20.20	19.0	0.57	0.079

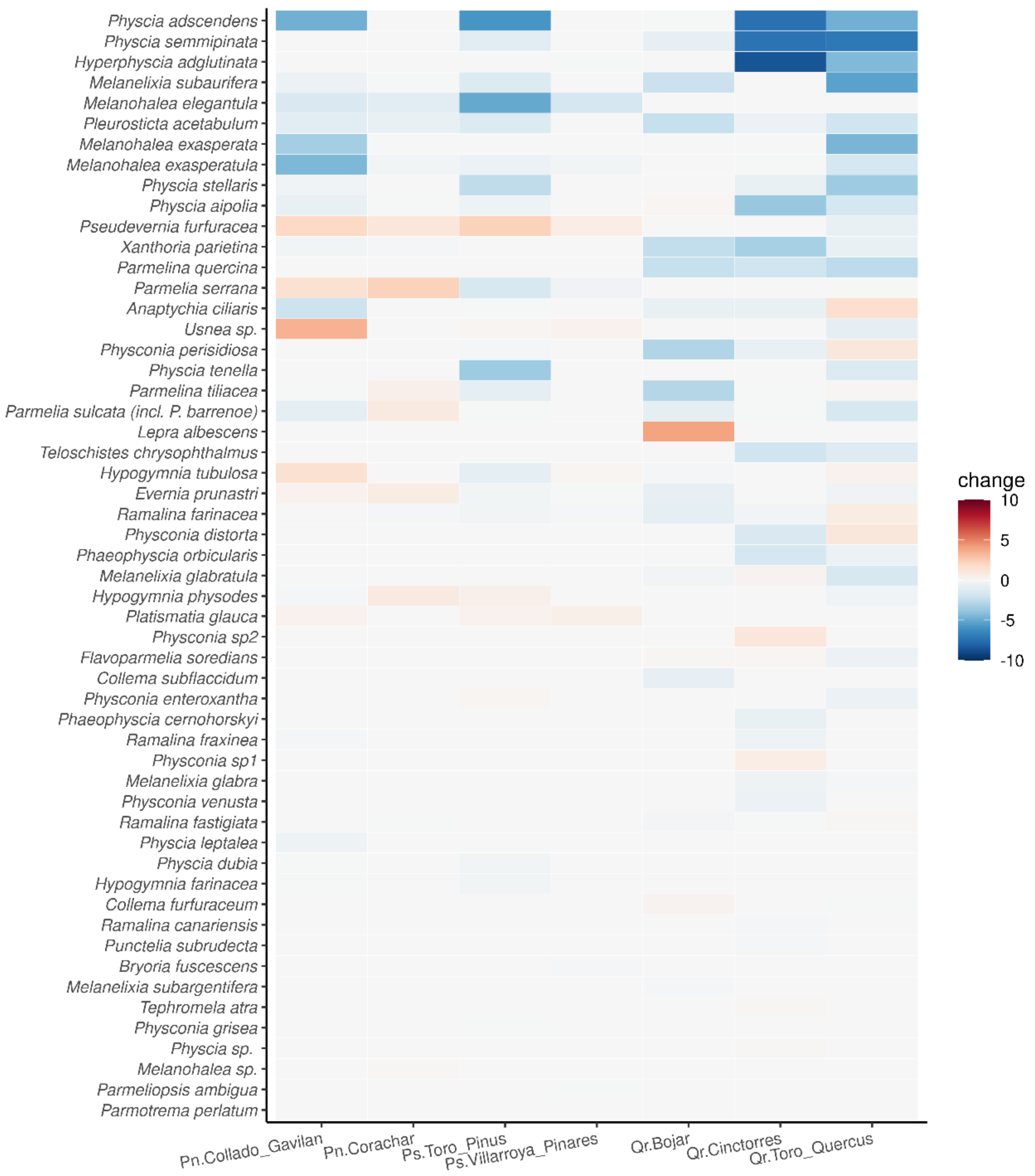

Figure S3: Mean change in abundance for each locality and species from 1997 to 2022. Blue color means the species abundance decreased while red color means an increase in abundance. Localities are grouped by phorophyte (Pn: *Pinus nigra*, Ps: *Pinus sylvestris*, Qr: *Quercus*) and species are ordered from larger to lower absolute change.

Table S5: Mean Damage Index (DI) values for each locality, Student's test and resdiuals Shapiro test results.

**Locality**	**DI.1997**	**DI.2022**	**t.test.p**	**Shapiro.p**
Bojar	2.47	2.32	0.2	1.8e-12
Cinctorres	1.93	2.25	0.0014	5.6e-17
Collado Gavilan	1.41	2.09	4.2e-12	2.4e-15
Corachar	4.63	2.48	1.9e-21	3.4e-06
Toro-*Pinus*	1.56	2.38	3.8e-17	2.1e-15
Toro-*Quercus*	1.28	2.14	1.3e-37	8.6e-21
Villarroya Pinares	1.98	2.33	0.035	1.9e-08

Figure S4: Effect of species characteristics solar irradiance, water requirement and poleotolerance in the abundance change between 1997 and 2022.

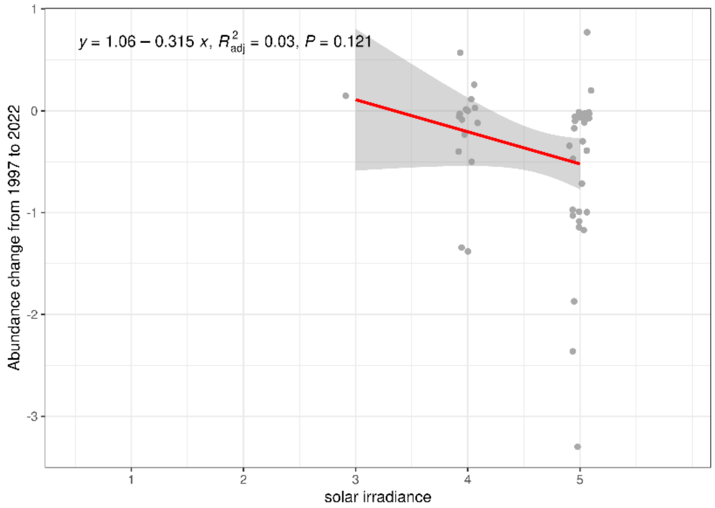

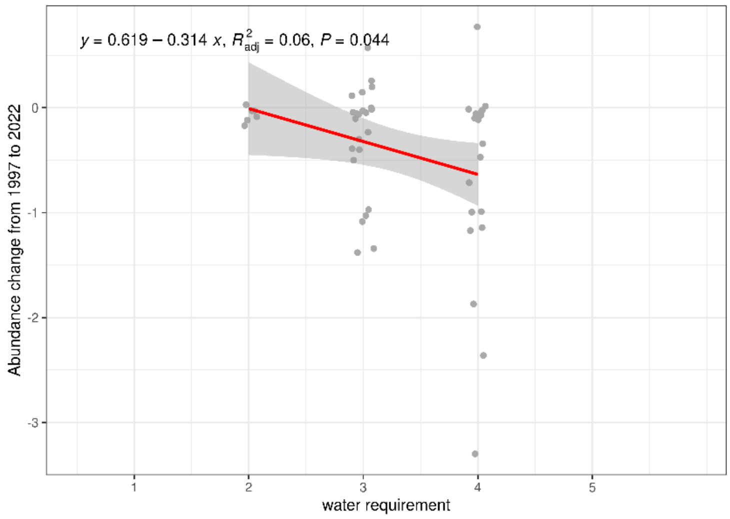

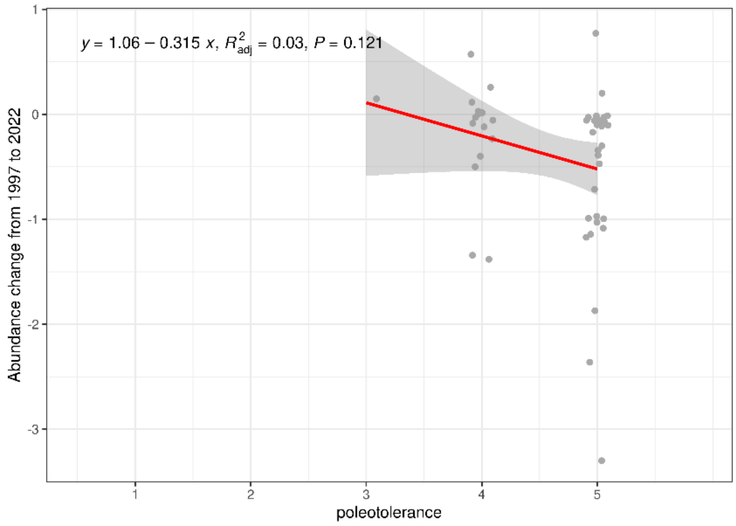

$y = 1.06 - 0.315\ x,\ R^2_{adj} = 0.03,\ P = 0.121$
Abundance change from 1997 to 2022
poleotolerance
1
0
-1
-2
-3
1
2
3
4
5

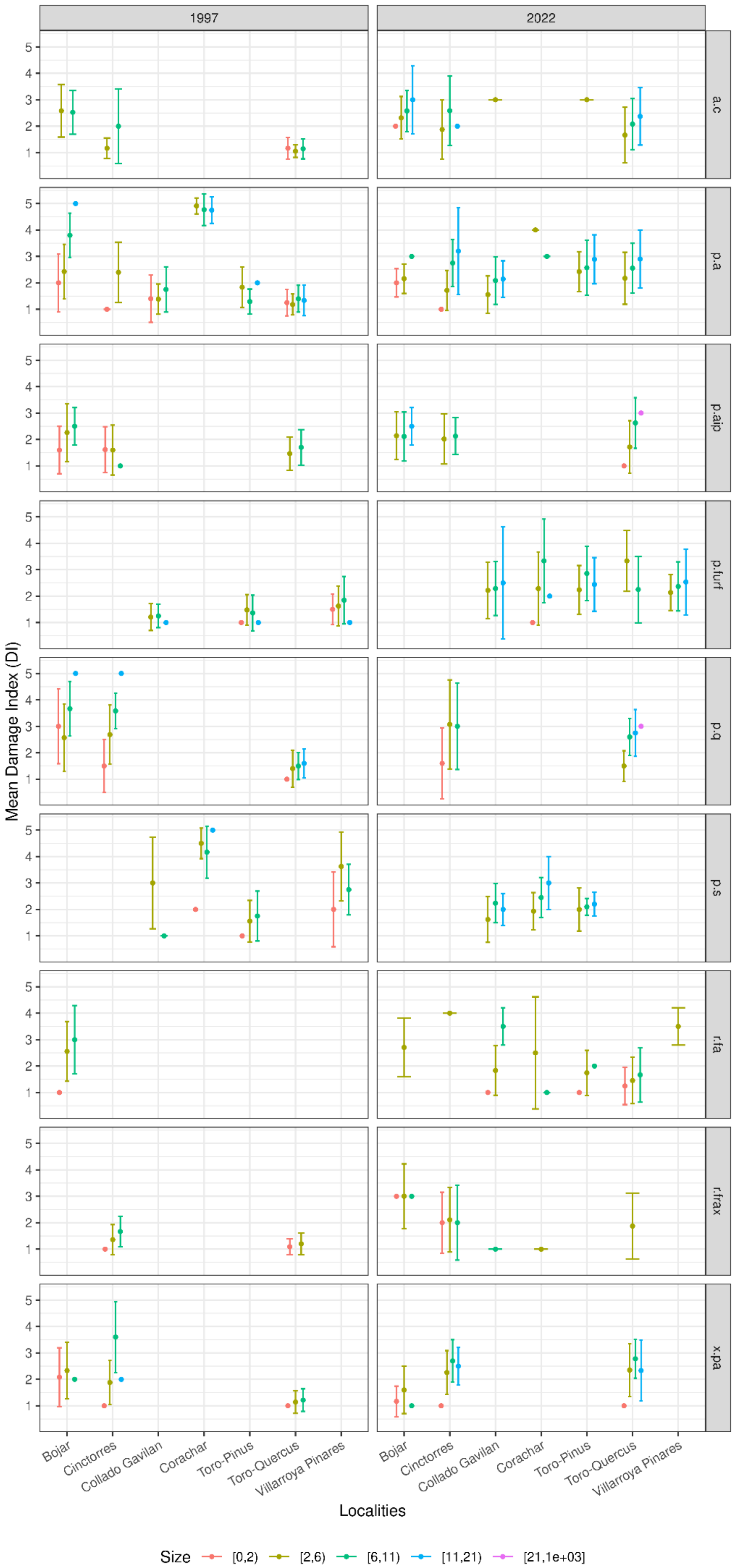
1997
2022
a.c
p.a
p.alp
p.furf
p.q
p.s
r.fa
r.frax
x.pa
Mean Damage Index (DI)
Bojar
Cinctorres
Collado Gavilan
Corachar
Toro-Pinus
Toro-Quercus
Villarroya Pinares
Localities
Size
[0,2)
[2,6)
[6,11)
[11,21)
[21,1e+03]

Figure S5: Mean Damage Index (ID) by size and species in each locality and year.

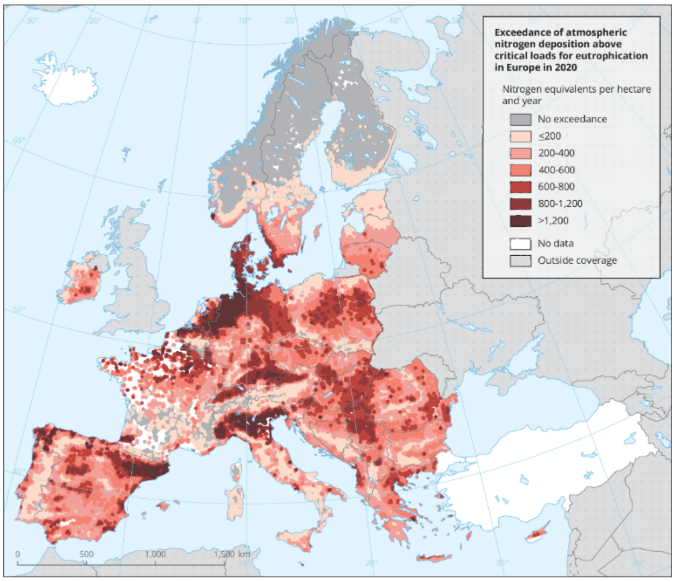

Reference data: ©ESRI

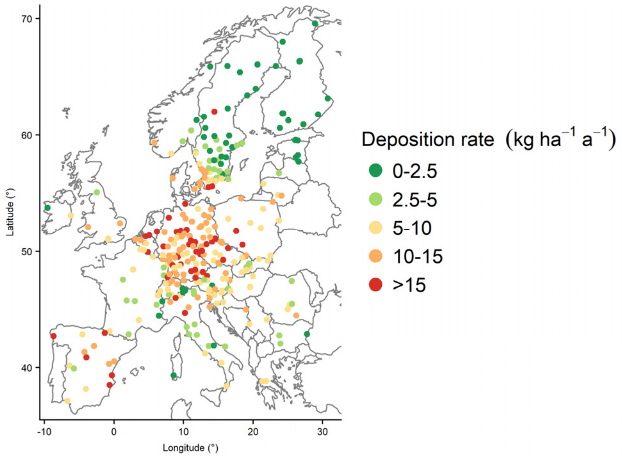

Figure S6: Exceedance of atmospheric nitrogen deposition above critical loads for eutrpphication in Europe in 2020. The map shows the ecosystem areas at risk of eutrophication in 2020. Critical loads refer to the upper limits of one or more pollutants deposited on the Earth's surface that an ecosystem, such as nutrient-poor grasslands or forests, can tolerate without its function (e.g. the nutrient nitrogen cycle) or its structure (e.g. plant species' richness) being damaged. If the deposition of airborne nitrogen (nitrate and ammonium compounds) is in excess of these critical loads, this is termed an 'exceedance', and an ecosystem is considered at risk of eutrophication. The map shows areas where critical loads are not exceeded (grey shading), indicating no risk of eutrophication, and where atmospheric nitrogen deposition exceeds critical loads, by magnitude of exceedance. (https://www.eea.europa.eu/data-and-maps/figures/exceedance-of-atmospheric-nitrogen-deposition)
