# Supplementary material for: Lichen Biodiversity and Near-Infrared Metabolomic Fingerprint as Diagnostic and Prognostic Complementary Tools for Biomonitoring: A Case Study in the Eastern Iberian Peninsula
Source: J Fungi (Basel). 2023 Oct 31;9(11):1064. doi: 10.3390/jof9111064 (PMC10672448; doi:10.3390/jof9111064)
Supplement: Supplementary file 1 [file jof-09-01064-s001.zip › Tables S1 and S2.pdf]

Locality code	Country, Province	masl	Coordinates	Phorophyte	Potential vegetation	Bioclimatic belt
**Toro_*Pinus***	Spain, Castellón, El Toro	1480	39.903497, -0.808658	*Pinus sylvestris*	*Junipero thuriferae-Querceto rotundifoliae sigmetum*	Subhumid Supramediterranean
**Toro_*Quercus***	Spain, Castellón, El Toro	1240	39.955159, -0.785412	*Quercus rotundifolia*	*Junipero thuriferae-Querceto rotundifoliae sigmetum*	Subhumid Supramediterranean
**Cinctorres**	Spain, Castellón, Morella	940	40.602866, -0.143989	*Quercus rotundifolia*	*Junipero thuriferae-Querceto rotundifoliae sigmetum*	Arid Supramediterranean
**Corachar**	Spain, Castellón, Corachar	1200	40.683990, 0.076189	*Pinus nigra*	*Junipero thuriferae-Querceto rotundifoliae sigmetum*	Arid Supramediterranean
**Bojar**	Spain, Castellón, Bojar	1100	40.679933, 0.108918	*Quercus rotundifolia*	*Violo willkommii-Querceto fagineae sigmetum*	Subhumid Supramediterranean
**Collado_Gavilán**	Spain, Teruel, Puebla de Valverde	1500	40.17179, -1.01613	*Pinus nigra*	*Junipereto hemisphaerico-thuriferae sigmetum*	Arid Supramediterranean
**Villarroya_Pinares**	Spain, Teruel, Fortanete	1700	40.509527, -0.614385	*Pinus sylvestris*	*Sabino-Pineto sylvestris sigmetum*	Arid Supramediterranean

Table S1. Characteristics of the seven biomonitoring plots included in this study.

	0	1	2	3	4	5
**tolerance to eutrophication**		no eutrophication	very weak eutrophication	weak eutrophication	rather high eutrophication	very high eutrophication
**water requirement**		hygrophytic	rather hygrophytic	mesophytic	xerophytic	very xerophytic
**pH of the substratum**		very acid	acid	subacid to subneutral	slightly basic	basic
**solar irradiance**		very shaded situations	shaded situations	plenty of diffuse light but scarce direct solar irradiation	sun-exposed sites, but avoiding extreme solar irradiation	very high direct solar irradiation
**poleotolerance**	species which exclusively occur on old trees in ancient, undisturbed forests	species occurring in natural or semi-natural habitats	species occurring in moderately disturbed areas	species occurring in heavily disturbed areas		

Table S2. Functional traits categories proposed by Nimis (2023) used in this study
